# Sarcopenia: Uma Importante Entidade ainda pouco Investigada na Insuficiência Cardíaca

**DOI:** 10.36660/abc.20230387

**Published:** 2023-07-27

**Authors:** Roberto Magalhães Saraiva, Andréa Rodrigues da Costa

**Affiliations:** 1 Instituto Nacional de Infectologia Evandro Chagas Fundação Oswaldo Cruz Rio de Janeiro RJ Brasil Instituto Nacional de Infectologia Evandro Chagas/Fundação Oswaldo Cruz, Rio de Janeiro, RJ – Brasil

**Keywords:** Sarcopenia, Insuficiência Cardíaca, Envelhecimento

A sarcopenia é considerada uma síndrome dependente da idade, caracterizada por declínio gradual da massa muscular esquelética, força e desempenho físico, sendo reconhecida como entidade clínica específica pela Organização Mundial da Saúde apenas em 2016. Os critérios diagnósticos têm sido motivo de debate. No entanto, o European Working Group on Sarcopenia in Older People (EWGSOP), em seu consenso revisado,^1^ definiu que o diagnóstico de sarcopenia deve começar com baixa força muscular como primeiro critério para identificar pacientes com provável sarcopenia, o que somado ao critério de baixa quantidade/qualidade muscular confirmaria o diagnóstico de sarcopenia, e somado ao baixo desempenho físico confirmaria grave sarcopenia. Os diferentes métodos e seus pontos de corte para avaliar massa muscular, força e capacidade de exercício para homens e mulheres foram recentemente revisados em outro trabalho.^2^

A sarcopenia piora com a idade; espera-se uma perda anual de 1-2% na massa muscular após os 50 anos^3^ e um aumento anual de 3% na prevalência de sarcopenia após os 60 anos.^4^ No entanto, pacientes com insuficiência cardíaca (IC) apresentam maior prevalência de sarcopenia do que seus congêneres com idade semelhante, mas sem IC.^5^ Uma metanálise recente descreveu que a prevalência de sarcopenia em pacientes com IC foi de 31%.^6^ De fato, as vias da fisiopatologia da sarcopenia e da IC compartilham componentes importantes, como alterações hormonais, inflamação, estresse oxidativo, apoptose e superativação do sistema ubiquitina-proteassoma, e alguns aspectos da IC, como baixo fluxo sanguíneo muscular, podem favorecer a sarcopenia,^7^ o que pode explicar a maior prevalência de sarcopenia em pacientes com IC e seu pior prognóstico.^8 , 9^ No entanto, há pouca evidência sobre intervenções terapêuticas para tratar a sarcopenia em pacientes com IC. Abordagens farmacológicas, nutricionais, hormonais e baseadas em exercícios foram postuladas como benéficas. Os inibidores da enzima conversora de angiotensina (ECA) e os bloqueadores dos receptores da angiotensina (BRA) demonstraram ter propriedades de proteção muscular em estudos pré-clínicos,^10^ mas os estudos clínicos falharam em provar que essas drogas podem melhorar a distância percorrida ou a força e função muscular em idosos.^11^ A espironolactona pode retardar o progresso da sarcopenia.^12^ A suplementação de vitamina D aumenta a força muscular esquelética em idosos^13^ e pode ajudar pacientes com IC sarcopênica. Outra terapia potencial poderia ser a terapia de suplementação de testosterona para atingir a faixa fisiológica, mas sua segurança em pacientes com IC ainda não foi testada. Uma terapia que combine suplementação nutricional e reabilitação cardíaca, incluindo exercícios, é a melhor abordagem para tratar a sarcopenia.^14 , 15^

O artigo publicado nesta edição dos Arquivos Brasileiros de Cardiologia traz informações sobre os pacientes brasileiros com IC, especificamente idosos ambulatoriais. Entre eles, os que tinham sarcopenia eram mais velhos, tinham menor índice de massa corporal (IMC), menor fração de ejeção (FE) do ventrículo esquerdo, pior capacidade funcional e pior qualidade de vida. É importante ressaltar que, após ajuste para idade, IMC, etnia, FE do ventrículo esquerdo e uso de inibidores da ECA/BRA, os pacientes com sarcopenia apresentaram níveis séricos de IL-6 mais elevados e pior capacidade funcional. Nessa população, sarcopenia ou sarcopenia grave foi diagnosticada em 26 pacientes (28,8%). Este estudo é muito importante, pois descreve uma alta prevalência de sarcopenia e como isso afeta a qualidade de vida desses pacientes.^16^ A sarcopenia ainda é pouco investigada e subtratada na IC e merece mais atenção e estudos clínicos por ser um potencial alvo de tratamento.

## References

[1] Cruz-Jentoft AJ, Bahat G, Bauer J, Boirie Y, Bruyère O, Cederholm T (2019). Sarcopenia: Revised European Consensus on Definition and Diagnosis. Age Ageing.

[2] Bielecka-Dabrowa A, Ebner N, Santos MR, Ishida J, Hasenfuss G, von Haehling S (2020). Cachexia, Muscle Wasting, and Frailty in Cardiovascular Disease. Eur J Heart Fail.

[3] Dodds RM, Granic A, Davies K, Kirkwood TB, Jagger C, Sayer AA (2017). Prevalence and Incidence of Sarcopenia in the Very Old: Findings from the Newcastle 85+ Study. J Cachexia Sarcopenia Muscle.

[4] Evans W (1997). Functional and Metabolic Consequences of Sarcopenia. J Nutr.

[5] Fülster S, Tacke M, Sandek A, Ebner N, Tschöpe C, Doehner W (2013). Muscle Wasting in Patients with Chronic Heart Failure: Results from the Studies Investigating Co-Morbidities Aggravating Heart Failure (SICA-HF). Eur Heart J.

[6] Chen R, Xu J, Wang Y, Jiang B, Xu X, Lan Y (2023). Prevalence of Sarcopenia and its Association with Clinical Outcomes in Heart Failure: An Updated Meta-Analysis and Systematic Review. Clin Cardiol.

[7] Curcio F, Testa G, Liguori I, Papillo M, Flocco V, Panicara V (2020). Sarcopenia and Heart Failure. Nutrients.

[8] Onoue Y, Izumiya Y, Hanatani S, Tanaka T, Yamamura S, Kimura Y (2016). A Simple Sarcopenia Screening Test Predicts Future Adverse Events in Patients with Heart Failure. Int J Cardiol.

[9] Nozaki Y, Yamaji M, Nishiguchi S, Fukutani N, Tashiro Y, Shirooka H (2019). Sarcopenia Predicts Adverse Outcomes in an Elderly Outpatient Population with New York Heart Association Class II–IV Heart Failure: A Prospective Cohort Study. Aging Med Healthcare.

[10] Anker SD, Negassa A, Coats AJ, Afzal R, Poole-Wilson PA, Cohn JN (2003). Prognostic Importance of Weight Loss in Chronic Heart Failure and the Effect of Treatment with Angiotensin-Converting-Enzyme Inhibitors: An Observational Study. Lancet.

[11] Zhou LS, Xu LJ, Wang XQ, Huang YH, Xiao Q (2015). Effect of Angiotensin-Converting Enzyme Inhibitors on Physical Function in Elderly Subjects: A Systematic Review and Meta-Analysis. Drugs Aging.

[12] Burton LA, McMurdo ME, Struthers AD (2011). Mineralocorticoid Antagonism: A Novel Way to Treat Sarcopenia and Physical Impairment in Older People?. Clin Endocrinol.

[13] Beaudart C, Buckinx F, Rabenda V, Gillain S, Cavalier E, Slomian J (2014). The Effects of Vitamin D on Skeletal Muscle Strength, Muscle Mass, and Muscle Power: a Systematic Review and Meta-Analysis of Randomized Controlled Trials. J Clin Endocrinol Metab.

[14] Fernández-Pombo A, Rodríguez-Carnero G, Castro AI, Cantón-Blanco A, Seoane LM, Casanueva FF (2021). Relevance of Nutritional Assessment and Treatment to Counteract Cardiac Cachexia and Sarcopenia in Chronic Heart Failure. Clin Nutr.

[15] Cho J, Choi Y, Sajgalik P, No MH, Lee SH, Kim S (2020). Exercise as a Therapeutic Strategy for Sarcopenia in Heart Failure: Insights into Underlying Mechanisms. Cells.

[16] Sangali TD, Souza GC, Ribeiro ECT, Perry IDS (2023). Sarcopenia: Marcadores Inflamatórios e Humorais em Pacientes Idosos com Insuficiência Cardíaca. Arq Bras Cardiol.

