# Supplementary material for: Sarcopenia: Uma Importante Entidade ainda pouco Investigada na Insuficiência Cardíaca
Source: Arq Bras Cardiol. 2023 Jul 27;120(7):e20230387. [Article in Portuguese] doi: 10.36660/abc.20230387 (PMC10382147; doi:10.36660/abc.20230387)
Supplement: Translation

# Translation: Sarcopenia: An Important Entity Still Underinvestigated in Heart Failure

**Authors:** Roberto Magalhães Saraiva, Andréa Rodrigues da Costa

**Affiliations:** 1 Brasil Instituto Nacional de Infectologia Evandro Chagas/Fundação Oswaldo Cruz, Rio de Janeiro, RJ – Brasil

**Keywords:** Sarcopenia, Heart Failure, Aging

Sarcopenia is considered an age-dependent syndrome characterized by a gradual decline in skeletal muscle mass, strength, and physical performance that was recognized as a specific clinical entity by the World Health Organization only in 2016. The diagnostic criteria have been a matter of debate. However, the European Working Group on Sarcopenia in Older People (EWGSOP), in their revised consensus,^1^ defined that sarcopenia diagnosis must start with low muscle strength as the first criterion to identify patients with probable sarcopenia, which added to low muscle quantity/quality criterion would confirm the diagnosis of sarcopenia, and added to low physical performance would confirm severe sarcopenia. The different methods and their cut-offs to evaluate muscle mass, force, and exercise capacity for men and women were recently reviewed elsewhere.^2^

Sarcopenia worsens with age; an annual loss of 1-2% in muscle mass is expected after 50 years old,^3^ and an annual 3% increase in sarcopenia prevalence occurs after 60 years old.^4^ However, patients with heart failure (HF) present a higher prevalence of sarcopenia than their counter mates with similar age but no HF.^5^ A recent meta-analysis described that the prevalence of sarcopenia in HF patients was 31%.^6^ In fact, the pathophysiology pathways of sarcopenia and HF share important components, such as hormonal changes, inflammation, oxidative stress, apoptosis, and overactivation of the ubiquitin-proteasome system, and some aspects of HF, such as low muscle blood flow, may favor sarcopenia,^7^ which may explain the higher prevalence of sarcopenia in HF patients and their poorer prognosis.^8 , 9^ However, there is little evidence on therapeutical interventions to address sarcopenia in HF patients. Pharmacological, nutritional, hormonal, and exercise-based approaches have been postulated to be beneficial. Angiotensin-converting enzyme (ACE) inhibitors and angiotensin receptor blockers (ARBs) have been shown to have muscle-protective properties in pre-clinical studies,^10^ but clinical studies failed to prove that these drugs can improve walk distance or muscle strength and function in the elderly.^11^ Spironolactone may delay sarcopenia progress.^12^ Vitamin D supplementation increases skeletal muscle strength in elderly subjects^13^ and may help sarcopenic HF patients. Another potential therapy could be testosterone supplementation therapy to reach the physiological range, but its safety in HF patients is yet to be tested. A therapy combining nutritional supplementation and cardiac rehabilitation, including exercise, is the best approach to treat sarcopenia.^14 , 15^

The article published in this issue of the *Arquivos Brasileiros de Cardiologia* adds information about Brazilian HF patients, specifically ambulatory elderly patients. Among them, those with sarcopenia were older, had lower body mass index (BMI), lower left ventricular ejection fraction (EF), worse functional capacity, and worse quality of life. Importantly, after adjusting for age, BMI, ethnicity, left ventricular EF, and use of ACE inhibitors/ARBs, patients with sarcopenia had higher IL-6 serum levels and worse functional capacity. In this population, sarcopenia or severe sarcopenia was diagnosed in 26 patients (28.8%). This study is very important as it describes a high prevalence of sarcopenia and how it affects the quality of life in these patients.^16^ Sarcopenia is still underinvestigated and undertreated in HF and deserves more attention and clinical studies as it is a potential target for treatment.
